# Low nuclear expression of HIF‐hydroxylases PHD2/EGLN1 and PHD3/EGLN3 are associated with poor recurrence‐free survival in clear cell renal cell carcinoma

**DOI:** 10.1002/cam4.6998

**Published:** 2024-02-24

**Authors:** Lassi Luomala, Kalle Mattila, Paula Vainio, Harry Nisén, Teijo Pellinen, Jouni Lohi, Teemu D. Laajala, Petrus Järvinen, Anna‐Riina Koskenniemi, Panu Jaakkola, Tuomas Mirtti

**Affiliations:** ^1^ Dept. of Urology Helsinki University Hospital and University of Helsinki Helsinki Finland; ^2^ Department of Oncology and Radiotherapy, FICAN West Cancer Centre University of Turku, Turku University Hospital Turku Finland; ^3^ InFlames Research Flagship University of Turku Turku Finland; ^4^ Dept. of Pathology, Turku University Hospital University of Turku Turku Finland; ^5^ Institute for Molecular Medicine Finland, Helsinki Institute of Life Science University of Helsinki Helsinki Finland; ^6^ Diagnostic Center, HUSLAB Laboratory Services Helsinki University Hospital and University of Helsinki Helsinki Finland; ^7^ Research Program in Systems Oncology (ONCOSYS) and iCAN – Digital Precision Cancer Medicine Flagship University of Helsinki Helsinki Finland; ^8^ Foundation for the Finnish Cancer Institute Helsinki Finland

**Keywords:** biomarker, hydroxylase‐domain proteins (PHD), hypoxia inducible factors (HIF), recurrence‐free survival, renal cell carcinoma (RCC)

## Abstract

**Background:**

Hypoxia inducible factors, HIF‐1α and HIF‐2α, and their main regulators, the prolyl hydroxylase domain proteins (PHDs), mediate cellular response to hypoxia and contribute to tumor progression in clear cell renal cell carcinoma (ccRCC). These biomarkers may improve the value of traditional histopathological features in predicting disease progression after nephrectomy for localized ccRCC and guide patient selection for adjuvant treatments.

**Patients and Methods:**

In this study, we analyzed the associations of PHD2 and PHD3 with histopathological tumor features and recurrence‐free survival (RFS) in a retrospective cohort of 173 patients who had undergone surgery for localized ccRCC at Helsinki University Hospital (HUH), Finland. An external validation cohort of 191 patients was obtained from Turku University Hospital (TUH), Finland. Tissue‐microarrays (TMA) were constructed using the primary tumor samples. Clinical parameters and follow‐up information from 2006 to 2019 were obtained from electronic medical records. The cytoplasmic and nuclear expression of PHD2, and PHD3 were scored based on immunohistochemical staining and their associations with histopathological features and RFS were evaluated.

**Results:**

Nuclear PHD2 and PHD3 expression in cancer cells were associated with lower pT‐stage and Fuhrman grade compared with negative nuclei. Patients with positive nuclear expression of PHD2 and PHD3 in cancer cells had favorable RFS compared with patients having negative tumors. The nuclear expression of PHD2 was independently associated with a decreased risk of disease recurrence or death from RCC in multivariable analysis. These results were observed in both cohorts.

**Conclusions:**

The absence of nuclear PHD2 and PHD3 expression in ccRCC was associated with poor RFS and the nuclear expression of PHD2 predicted RFS regardless of other known histopathological prognostic factors. Nuclear PHD2 and PHD3 are potential prognostic biomarkers in patients with localized ccRCC and should be further investigated and validated in prospective studies.

## Graphical abstract

The prolyl hydroxylase domain proteins (PHDs) mediate cellular response to hypoxia and contribute to tumor progression in clear cell renal cell carcinoma (ccRCC). In our externally validated study, we analyzed the association of PHD2&3 with tumor features and recurrence free survival (RFS). Our findings suggest, that PHD2 and PHD3 are associated with poor prognosis in clear cell renal cell carcinoma (ccRCC) and are valuable prognostic biomarkers in localized ccRCC.

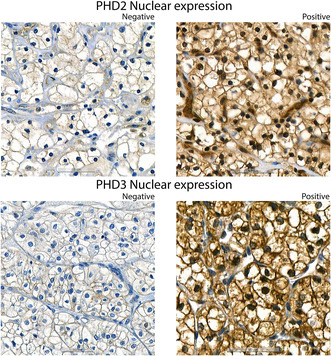

## 1 INTRODUCTION

Renal cell carcinoma (RCC) represents 2% of all cancer deaths worldwide.^1^ The primary treatment of localized RCC is radical or partial nephrectomy (PN). However, one third of the patients with localized RCC will eventually develop metastases after surgery.^2^ Metastases lead usually to death although antiangiogenic receptor tyrosine kinase inhibitors (TKI), immune checkpoint inhibitors (ICI), and combination therapies have prolonged survival in metastatic RCC.^3^ In addition, a recent Phase III trial led to the approval of pembrolizumab as an adjuvant treatment for patients with clear cell RCC (ccRCC) at intermediate‐high and high risk of recurrence after nephrectomy.^4^ However, the optimal selection of patients for adjuvant treatment is unclear as three other adjuvant and perioperative treatment trials failed to improve recurrence‐free survival (RFS).^5^, ^6^, ^7^

In current clinical practice, there are several prognostic models^2^, ^4^, ^8^, ^9^, ^10^ to assess the risk of metastases or death after surgery for localized RCC. These algorithms are based on clinical and histopathological variables, such as the tumor‐node‐metastasis (TNM) stage,^11^ Fuhrman grade,^12^ WHO/ISUP grade,^13^ sarcomatoid differentiation,^13^ and Eastern Cooperative Oncology Group performance status (ECOG PS).^14^ The prediction accuracy (concordance index) of these algorithms has generally exceeded 70% in patients with ccRCC.^15^ Since the introduction of effective systemic treatments and the availability of adjuvant treatment, there has been a need for individualized, risk‐based follow‐up with thoracic and abdominal computed tomography (CT) scans and improved selection of patients for adjuvant treatment after surgery for localized RCC. Novel tumor tissue biomarkers could provide additional prognostic information and have been extensively studied in metastatic RCC but, so far, none of them has been adopted into routine clinical practice.^16^

Insufficient oxygen availability, hypoxia, is common in RCC. Hypoxia inducible factors HIF‐1α and HIF‐2α (HIFs) and their regulators, the prolyl hydroxylase domain proteins that is, HIF prolyl hydroxylases (PHDs), play a central role in mediating cellular response to hypoxia and contribute to progression of RCC.^17^, ^18^ In the presence of sufficient oxygen, PHDs hydroxylate HIF‐α subunits at two proline residues. This leads to binding of Von Hippel Lindau (VHL) E3‐ubiquitin ligase tumor suppressor protein and subsequent increase in HIF‐α ubiquitination and proteasomal degradation. The biallelic loss or inactivation of VHL gene is typical in ccRCC.^19^ Hypoxia, or the inactivation of VHL independent of tissue oxygen levels, results in the accumulation of HIF‐1α and HIF‐2α transcription factors in cancer cells. This affects the expression of over 300 genes regulating angiogenesis, cell cycle, and tumor metabolism.^17^ Among the activated genes are PHD2 (a.k.a. EGLN1) and PHD3 (a.k.a. EGLN3) which generate a negative feedback loop in normal conditions.^20^ PHD2 is the main regulator of HIF‐1α stability whereas HIF‐2α isoform is generally thought to be mainly regulated by PHD3.^21^ In addition, PHD2 and, in particular PHD3, have been suggested to possess other tumor suppressor properties apart from the HIF signaling pathway.^22^, ^23^

The prognostic value of biomarkers in the HIF pathway is controversial, indicating that the signaling network may be more complex than previously thought. There are several studies about HIF‐1α and HIF‐2α suggesting HIF‐1α to be associated with favorable prognosis and HIF‐2α with poor prognosis.^24^, ^25^ PHD3 has been reported to be inversely associated with Fuhrman grade^26^ but the evidence of prognostic potential of PHD2 and PHD3 in large cohort studies remains sparse. Although PHD2 is widely recognized as a key oxygen sensor regulating the HIF pathway and thereby inhibiting angiogenesis, the role of PHD2 in tumor suppression and as a prognostic factor is unclear.^22^, ^27^ Our aim was to study the associations of HIF‐hydroxylases, PHD2 and PHD3, with histopathological features and RFS in two separate cohorts of patients with localized ccRCC and to evaluate their potential as prognostic biomarkers.

## 2 PATIENTS AND METHODS

### 2.1 Helsinki training cohort

Electronic medical records were searched for patients treated with radical nephrectomy (RN) or PN at the Helsinki University Hospital (HUH) between 2006 and 2013 resulting in an initial cohort of 1223 patients with RCC. Patients with cytoreductive nephrectomy (M1), regional lymph node metastases (N1), previous kidney cancer in history, or multiple kidney tumors at the time of diagnosis, as well as patients with missing clinical data were excluded. The baseline prognostic features of the primary tumor including histological subtype, tumor size, TNM stage (according to AJCC 8th edition), tumor grade (determined using the 4‐tiered nuclear grading system applied at the time of diagnosis), information on the presence of micro‐ and macrovascular invasion, rhabdoid and sarcomatoid differentiation, histological tumor necrosis, a positive surgical margin, and tumor invasion into the adjacent structures were retrospectively obtained from the medical records and pathology reports of HUH. Histological features were centrally re‐assessed by two uropathologists (T.M. and J.L.). The baseline clinical features included age, sex, clinical stage determined with computational tomography (CT), and serum creatinine at the time of surgery.

**FIGURE 1:**
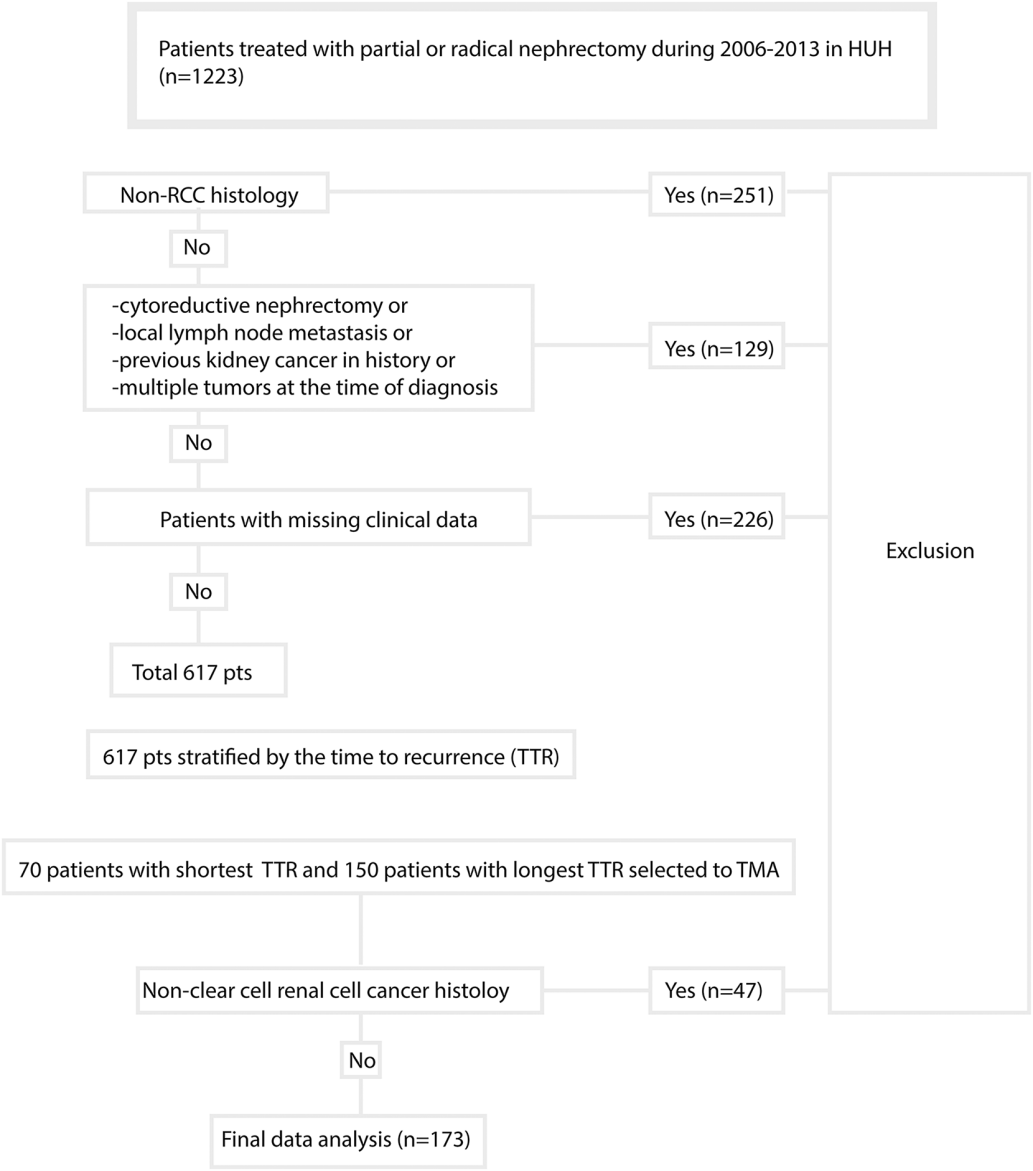
Patient selection criteria flow chart for Helsinki training cohort. HUH, Helsinki University Hospital.

Follow‐up information including the date of disease recurrence, death, or the last follow‐up visit and the cause of death (RCC or other) was obtained from the electronic medical records of HUH and the database of Statistics Finland. Postoperative follow‐up with regular thoracic and abdominal CT was performed according to local clinical practice to detect disease recurrence. The follow‐up cutoff date were September 9, 2019 for the training cohort. After analyzing RFS time, 70 patients with the shortest RFS and 150 patients with the longest RFS were selected for tissue microarray (TMA) construction. Patient selection criteria are described in Figure 1. A representative tissue block from each surgical specimen was selected by two pathologists (J.L. and T.M.). After excluding 47 patients with non‐clear cell histology, a total of 173 patients with localized ccRCC were included into the final TMA (Helsinki training cohort). The study protocol is reported according to the REMARK guideline for prognostic biomarkers.^28^

### 2.2 Turku validation cohort

An external validation cohort was obtained from Turku University Hospital, Finland using the same inclusion and exclusion criteria as for the Helsinki training cohort. The Turku validation cohort included 191 patients who had undergone PN or RN for localized (N0M0) ccRCC between 2005 and 2014 regardless of RFS time. A uropathologist (P.V.) selected the representative blocks from the surgical specimens for TMA construction and reviewed the original pathology reports. The baseline histopathological and clinical features as well as the follow‐up information for patients in the Turku validation cohort were collected (by K.M. and P.V.) from the electronic medical records of Turku University Hospital as described in the Helsinki training cohort. The follow‐up cutoff date were July 11, 2019 for the Turku validation cohort.

### 2.3 Tissue microarrays

Hematoxylin–eosin‐stained clinical surgical specimen microscope slides were digitally scanned for the selection of representative cancer and normal kidney tissue from the formalin‐fixed paraffin embedded (FFPE) tissue blocks, and for subsequent TMA annotation. For each slide, digital images were acquired at 0.24 μm/pixel resolution using a Pannoramic Flash III slide scanner (3DHistech, Budapest, Hungary). The images were uploaded to a secure server and annotated with Caseviewer software (3DHistech) for TMA construction with TMA Grand Master equipment (3DHistech). For each patient, two cores (1.0 mm in the Helsinki training cohort and 1.5 mm in the Turku validation cohort) from the central area of the tumor, two from tumor border area, and two representing adjacent normal kidney tissue were punched yielding altogether 1161 cores from the Helsinki training cohort and 1123 cores from the Turku validation cohort.

### 2.4 Immunohistochemistry

Sections of 3.5 μm were cut from the TMA blocks and mounted on adhesive microscope slides (Superfrost+, Menzel‐Glazer, Braunschweig, Germany). Immunohistochemical staining (IHC) was performed using an autostainer (Dako A/S, Glostrup, Denmark). First, TMA sections were deparaffinized and rehydrated. The IHC procedure included heat‐induced epitope retrieval in a pressure cooker using a citrate buffer (pH 6), followed by primary antibody incubation with the following antibodies and their respective dilutions: HIF‐1a (code 610959, BD Transduction Laboratories, 1:100), HIF‐2a (NB100‐122, NOVUS BIOLOGICALS, Cambridge, UK, 1:100), PHD2 (NB100‐137, NOVUS BIOLOGICALS, Cambridge, UK, 1:2000), and PHD3 (NB100‐139, NOVUS BIOLOGICALS, Cambridge, UK, 1:500). The primary antibodies were detected with Dako Envision anti‐rabbit/mouse HRP‐conjugated secondary antibodies (code K5007, Dako A/S, Glostrup, Denmark). All reactions were visualized using diaminobenzidine and the slides were counterstained with hematoxylin.

### 2.5 Scoring of protein expression

TMA slides were scanned with Pannoramic Flash III scanner and uploaded to Aiforia platform (Aiforia Technologies Plc., Helsinki, Finland). Samples were visually scored unaware of clinical data using Aiforia Cloud software (Aiforia Technologies Plc., Helsinki, Finland) in consensus by two observers (L.L., T.M.). For HIF‐1α, HIF‐2α, PHD2, and PHD3, the intensity of cytoplasmic or cell membrane staining (0 = no expression; 1 = weak expression; 2 = moderate expression; 3 = strong expression) in cancer cells, referred as cytoplasmic expression, was recorded and nuclear expression was recorded as positive (1) or negative (0) staining. Any nuclear staining visually observed in cancer cells was considered a positive result. The percentage of the cells demonstrating nuclear staining (nuclear percentage) was also recorded. Maximum values of four cores from each tumor were used in the final analysis.

### 2.6 Statistical analysis

Descriptive statistics included median with range or interquartile range (IQR) for continuous variables and frequency with percentages for categorical variables. Continuous variables were compared using the Kruskal–Wallis test and pairwise comparisons were performed using the Mann–Whitney‐*U* test. Categorical variables were compared using the Fisher exact test or Pearson's chi‐squared test. Spearman's rank order correlation was used to examine the associations between clinicopathological variables and HIF pathway proteins. The Kaplan–Meier method was used to illustrate the association of biomarkers with RFS. RFS was defined as the time from surgery to radiologically verified disease recurrence (event) or death from RCC (event). Patients who were alive or had died from other causes than RCC were censored at the time of the last follow‐up visit or death. The association of prognostic factors with RFS was evaluated using univariable Cox proportional hazards model followed by multivariable Cox proportional hazards model. *p* < 0.05 was considered significant. All analyses were performed with IBM SPSS (version 28) and R statistical software (version 4.3.0; R Core Team (2022). R: A language and environment for statistical URL https://www.R‐project.org/).

## 3 RESULTS

### 3.1 Clinical characteristics and treatment outcomes

Baseline patient and tumor characteristics at the time of surgery and treatment outcomes during the follow‐up period are described in Table 1. Patients in the Helsinki training cohort were on average 3 years younger (*p* = 0.01) and had more commonly pT3–pT4 tumors (*p* < 0.001) compared to patients in the Turku validation cohort. Histological tumor necrosis was more common (*p* = 0.009) in the Turku validation cohort.

**TABLE 1:** Patient characteristics.

Helsinki training cohort			Turku validation cohort		
	*n* = 173	%	*n* = 191	%	*p*‐value
Age, years					**0.01** (Kruskall–Wallis)
Median	65.567		68.054		
Range	23.96–89.62		33.64–90.22		
Sex					
Male	91	52.6	111	58.1	0.294 (Fisher)
Female	82	47.4	80	41.9	
2002 primary tumor classification					**<0.001** (Fisher)
pT1	79	45.7	116	60.7	
pT2	23	13.3	50	26.2	
pT3‐pT4	71	41.0	25	13.1	
Nuclear grade					0.664 (Fisher)
I	10	5.8	12	6.3	
II	99	57.2	97	50.8	
III	54	31.2	70	36.6	
IV	10	5.8	12	6.3	
Microvascular invasion	36	20.8	38	19.9	0.896 (Fisher)
Tumor necrosis	50	28.9	81	42.6	**0.009** (Fisher)
Sarcomatoid features	10	5.8	11	5.8	0.993 (Fisher)
Disease recurrence or death from RCC	70	40.5	68	35.6	0.331 (Fisher)
Death					**<0.001** (Fisher)
From RCC	46	26.6	56	9.3	
Other cause	10	5.8	40	20.9	
Missing cause of death	0	0	6	3.1	
Median follow‐up (IQR)	122.6 (109.2–131.4)		127.4 (106.3–157.8)		0.055 (Mann–Whitney)

Bold values were considered statistically significant at the *p* < 0.05 level.

After the median follow‐up of over 10 years in both cohorts, 72 patients (42%) in the Helsinki training cohort and 68 patients (36%) in the Turku validation cohort were diagnosed with disease recurrence or had died from RCC without statistically significant differences in the RF status between the patient cohorts (Table 1). In the Turku validation cohort, more patients had died from non‐RCC causes compared to the Helsinki training cohort (*p* < 0.001). There were six patients with unknown causes of death in the Turku validation cohort (of whom two patients had experienced disease recurrence earlier). Thus, RFS status could not be confirmed for four patients.

### 3.2 Expression of HIF pathway biomarkers

Positive nuclear expression of PHD2 and PHD3 in cancer cells was detected in 98.8% and 75.1% of patients in the Helsinki training cohort and in 72.2% and 44.3% of patients in the Turku validation cohort, respectively. The mean nuclear percentage of PHD2 and PHD3 was 55.0% (IQR 26.6%–85.0%) and 33.9% (IQR 0.6%–59.4%) of all nuclei in the Helsinki training cohort and 29.66% (IQR 0%–50%) and 8.0% (IQR 0%–5%) of all nuclei in the Turku cohort, respectively. Any cytoplasmic expression of PHD2 and PHD3 (weak, moderate, or strong) in cancer cells was observed in 100% of patients in the Helsinki training cohort and 100% and 91.1% of patients in the Turku validation cohort, respectively.

**FIGURE 2:**
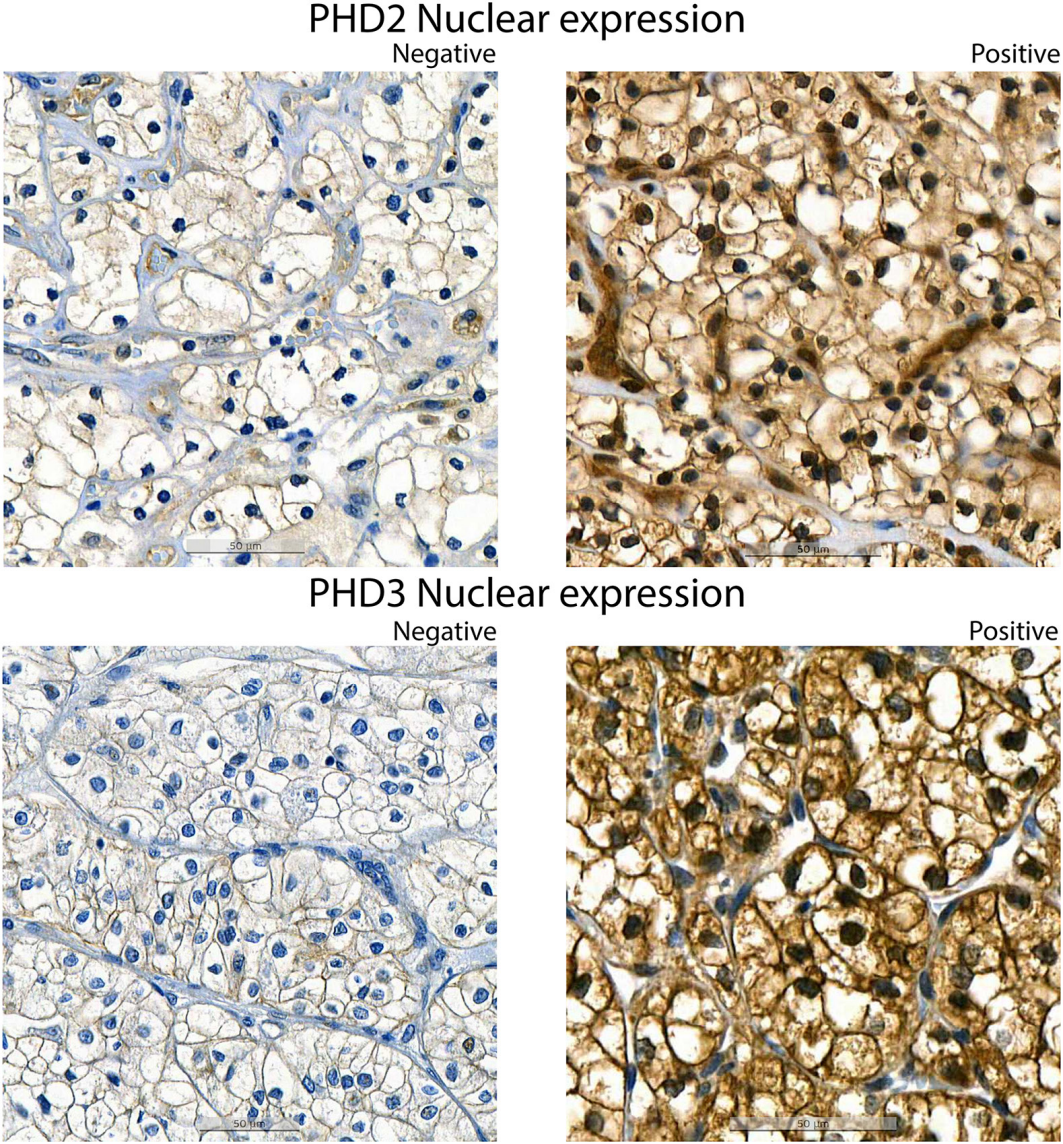
Representative images of nuclear expression (brown) of PHD2 and PHD3.

Representative images of nuclear PHD2 and PHD3 IHC staining are visualized in Figure 2. Examples of cytoplasmic PHD2 and PHD3 IHC staining are visualized in Figure S1. Distributions of the cytoplasmic expression of PHD2 and PHD3 (weak, moderate, and strong) and the nuclear expression of PHD2 and PHD3 (positive, negative) in both cohorts are described in Figure S2. Detailed information on the cytoplasmic and nuclear expression of HIF‐1α and HIF‐2α are described in Table S1. Distributions of HIF‐1α and HIF‐2α expressions are described in Figure S3 and examples of expression levels are shown in Figure S4.

### 3.3 Associations of HIF pathway biomarkers with tumor characteristics

Associations of nuclear expression of PHD2 and PHD3 with baseline clinical and histopathological features are detailed in Table 2. In the Helsinki training cohort, nearly all patients had positive nuclear PHD2 expression. Tumors with positive nuclear PHD3 expression had more commonly lower pT‐stage (*p* = 0.018) and lower nuclear grade (*p* = 0.019). In the Turku validation cohort, tumors with positive nuclear PHD2 expression had more commonly lower pT‐stage (*p* < 0.001), lower nuclear grade (*p* = 0.002), as well as lower rates of microvascular invasion (*p* < 0.001), histological tumor necrosis (*p* = 0.001), and sarcomatoid differentiation (*p* = 0.046). Tumors with positive nuclear PHD3 had lower rates of microvascular invasion (*p* = 0.005), histological tumor necrosis (*p* = 0.001), and sarcomatoid differentiation (*p* = 0.016) and statistically non‐significantly lower pT‐stage and lower nuclear grade.

**TABLE 2:** Associations of nuclear expression of PHD2 and PHD3 with baseline clinical and histopathological features and cytoplasmic expression of PHD2 and PHD3.

	Helsinki training cohort					
	Nuclear PHD2 negative *n* = 2 (1%)	Nuclear PHD2 positive *n* = 171 (99%)	*p*‐value	Nuclear PHD3 negative *n* = 43 (25%)	Nuclear PHD3 positive *n* = 130 (75%)	*p*‐value
pT1	1 (50%)	78 (46%)	0.236	12 (28%)	67 (52%)	**0.018**
pT2	1 (50%)	22 (13%)		6 (14%)	17 (13%)	
pT3–4	0 (0%)	71 (41%)		25 (58%)	46 (35%)	
Grade I	0 (0%)	10 (6%)	0.923	0 (0%)	10 (8%)	**0.019**
Grade II	1 (50%)	98 (57%)		20 (47%)	79 (61%)	
Grade III	1 (50%)	53 (31%)		18 (42%)	36 (28%)	
Grade IV	0 (0%)	10 (6%)		5 (12%)	5 (4%)	
Microvascular invasion	0 (0%)	36 (21%)	0.466	12 (28%)	24 (18%)	0.186
No microvascular invasion	2 (100%)	135 (79%)		31 (72%)	106 (82%)	
Tumor necrosis	0 (0%)	50 (29%)	0.364	16 (37%)	34 (26%)	0.166
No tumor necrosis	2 (100%)	121 (71%)		27 (63%)	96 (74%)	
Sarcomatoid features	0 (0%)	10 (6%)	0.725	5 (12%)	5 (4%)	0.058
No sarcomatoid features	2 (100%)	161 (94%)		38 (88%)	125 (96%)	

	Turku validation cohort					
	Nuclear PHD2 negative *n* = 54 (28%)	Nuclear PHD2 positive *n* = 137 (72%)	*p*‐value	Nuclear PHD3 negative *n* = 107 (56%)	Nuclear PHD3 positive *n* = 84 (44%)	*p*‐value
pT1	23 (43%)	93 (68%)	**<0.001**	61 (57%)	55 (66%)	0.096
pT2	17 (31%)	33 (24%)		27 (25%)	23 (27%)	
pT3–4	14 (26%)	11 (8%)		19 (18%)	6 (7%)	
Grade I	1 (2%)	11 (8%)	**0.002**	4 (4%)	8 (10%)	0.095
Grade II	18 (33%)	79 (58%)		50 (47%)	47 (56%)	
Grade III	30 (56%)	40 (29%)		44 (41%)	26 (31%)	
Grade IV	5 (9%)	7 (5%)		9 (8%)	3 (4%)	
Microvascular invasion	19 (35%)	19 (14%)	**<0.001**	29 (27%)	9 (11%)	**0.005**
No microvascular invasion	35 (65%)	118 (86%)		78 (73%)	75 (89%)	
Tumor necrosis	33 (61%)	48 (35%)	**0.001**	56 (53%)	25 (30%)	**0.001**
No tumor necrosis	21 (39%)	88 (65%)		50 (47%)	59 (70%)	
Sarcomatoid features	6 (11%)	5 (4%)	**0.046**	10 (9%)	1 (1%)	**0.016**
No sarcomatoid features	48 (89%)	132 (96%)		97 (91%)	83 (99%)	

*Note*: *N* = number of patients (with percentage), *p*‐values are from a chi‐squared test.

Bold values were considered statistically significant at the *p* < 0.05 level.

Spearman's rank order correlations were computed to analyze relationships between HIF pathway biomarkers and histopathological tumor characteristics. Statistically significant associations are shown in Table S2. Higher percentages of cancer cells with positive nuclear expression of PHD2 and PHD3 were associated with lower pT‐stage and lower nuclear grade in both cohorts. Higher cytoplasmic expression of PHD2 was associated with higher cytoplasmic expression of PHD3, HIF‐1α, and HIF‐2α in both cohorts. Higher cytoplasmic expression of PHD2 was associated with lower nuclear expression of HIF‐1α in both cohorts.

### 3.4 Association of HIF prolyl hydroxylases with RFS

**FIGURE 3:**
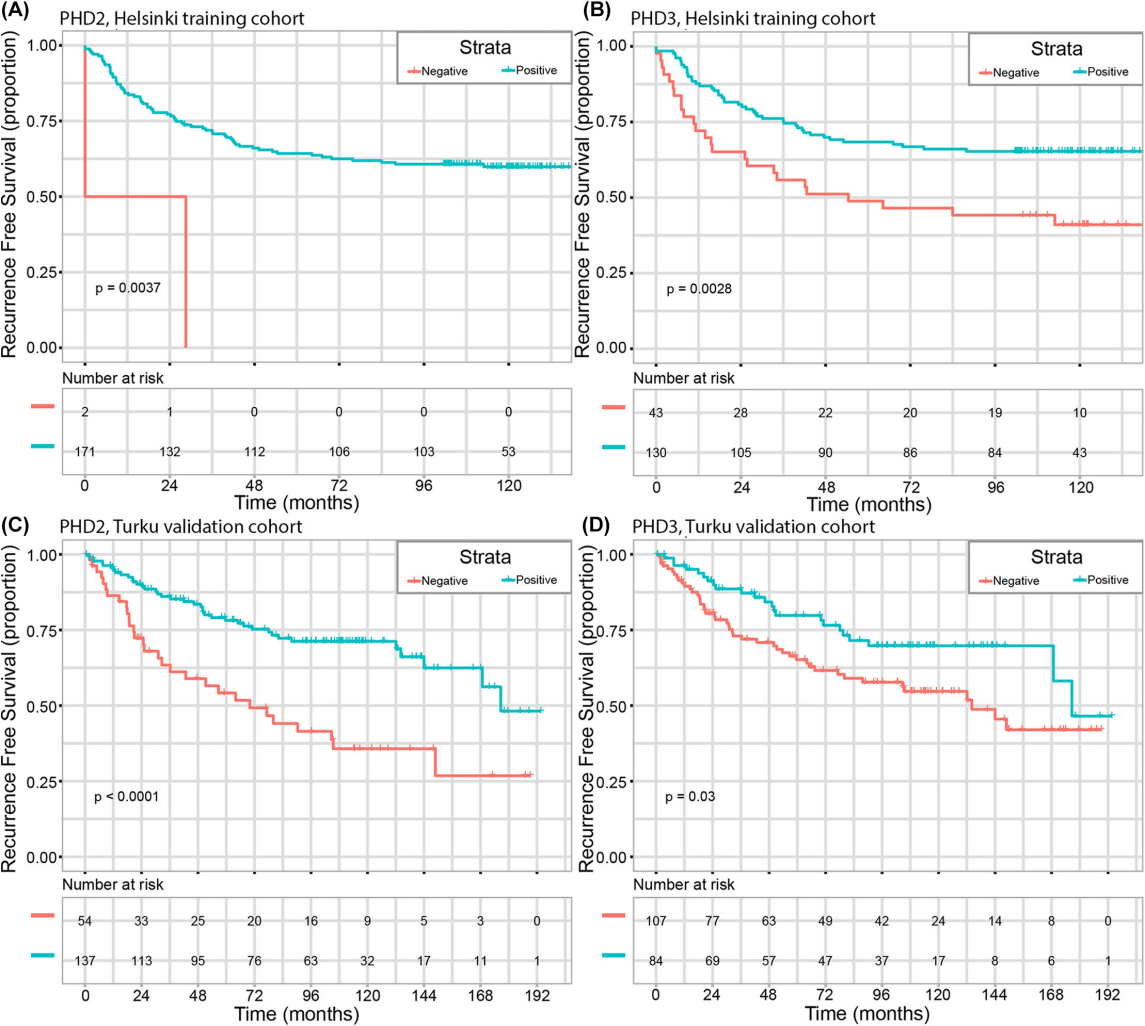
Kaplan–Meier survival analyses for probability of disease recurrence or death from RCC in the Helsinki training cohort and Turku validation cohort. PHD2 nuclear expression (A and C) and PHD3 nuclear expression (B and D) *P*‐values are from a two‐sided log‐rank (Mantel‐Cox) test.

Patients with positive nuclear expression of PHD2 in cancer cells as well as patients with positive nuclear expression of PHD3 in cancer cells had significantly longer RFS in both cohorts. Kaplan–Meier survival analyses for probability of disease recurrence or death from RCC are visualized in Figure 3. The median RFS (mRFS) of patients with positive nuclear PHD2 and PHD3 expression was not reached compared to the mRFS of 14.2 months in patients with negative nuclear PHD2 and 54.3 months in patients with negative nuclear PHD3 in the Helsinki training cohort (log‐rank *p* = 0.0037 and *p* = 0.0028, respectively). Similarly, median RFS times were longer in patients with positive nuclear PHD2 expression (mRFS 176.2 months) and PHD3 expression (mRFS 176.3 months) compared to patients with negative nuclear PHD2 expression (mRFS 69.9 months) and PHD3 expression (mRFS 133.8 months) in the Turku validation cohort (log‐rank *p* < 0.001 and *p* = 0.03, respectively). The patients without disease recurrence or death from RCC also had a higher nuclear percentage of PHD2 compared to patients with disease recurrence or death from RCC (*p* = 0.001 in the Helsinki cohort and *p* = 0.028 in the Turku cohort, Mann–Whitney *U*‐test) as visualized in Figure S5.

Cytoplasmic expressions of PHD2 or PHD3 were not associated with RFS in either of the cohorts. Cytoplasmic expressions of both HIF‐1α and HIF‐2α were significantly associated with shorter RFS in the Helsinki training cohort (log‐rank *p* = 0.004 and *p* < 0.001) but did not reach statistical significance in the Turku validation cohort (log‐rank *p* = 0.920 and *p* = 0.784). Kaplan–Meier survival analyses of HIF‐1α and HIF‐2α cytoplasmic expression are visualized in Figure S6. Nuclear expressions of HIF‐1α and HIF‐2α did not have significant associations with RFS.

### 3.5 Univariable and multivariable analyses of the association of patient and tumor characteristics with RF

We evaluated the prognostic value of the HIF prolyl hydroxylases PHD2, PHD3 and HIF‐1α, HIF‐2α, and the following clinical and histopathological features. The results of univariable and multivariable COX proportional hazards models in both cohorts are described in Table 3.

**TABLE 3:** Uni‐ and multivariable COX models for recurrence‐free survival.

Uni‐ and multivariable COX proportional hazards models for recurrence‐free survival									
Univariable COX model		Helsinki training cohort				Turku validation cohort			
Clinicopathological features		HR	Lower 95% CI	Upper 95% CI	*p*‐value	HR	Lower 95% CI	Upper 95% CI	*p*‐value
Age (years)		**1.028**	**1.006**	**1.050**	***0.011***	1.014	0.991	1.037	0.228
Sex	Male vs. female	1.186	0.754	1.866	0.461	1.342	0.822	2.191	0.239
pT‐stage	**Ref = Stage T1**	**1**			**<0.001**	**1**			**<0.001**
	**Stage T2**	**2.416**	**1.067**	**5.469**	**0.034**	**3.368**	**1.967**	**5.768**	**<0.001**
	**Stage T3–4**	**5.776**	**3.281**	**10.170**	**<0.001**	**3.387**	**1.747**	**6.566**	**<0.001**
Nuclear grade	**Ref = Grade I**	**1**			**<0.001**	**1**			**0.002**
	Grade II	1.781	0.422	7.521	0.432	1.568	0.372	6.610	0.540
	**Grade III**	**5.984**	**1.428**	**25.078**	***0.014***	3.426	0.821	14.300	0.091
	**Grade IV**	**11.154**	**2.390**	**52.047**	***0.002***	**5.568**	**1.151**	**26.920**	**0.033**
Peripelvic fat invasion	**Not present (ref) vs. present**	**2.32**	**1.419**	**3.792**	**<0.001**	**3.112**	**1.749**	**5.537**	**<0.001**
Perirenal fat invasion	**Not present (ref) vs. present**	**2.953**	**1.837**	**4.745**	**<0.001**	0.819	0.566	1.184	0.288
Macrovascular invasion	**Not present (ref) vs. present**	**3.516**	**2.127**	**5.812**	**<0.001**	**2.192**	**1.171**	**4.101**	**0.014**
Microvascular invasion	**Not present (ref) vs. present**	**3.936**	**2.433**	**6.367**	**<0.001**	**2.643**	**1.591**	**4.390**	**<0.001**
Tumor necrosis	**Not present (ref) vs. present**	**3.791**	**2.399**	**5.990**	**<0.001**	**4.822**	**2.861**	**8.126**	**<0.001**
Sarcomatoid features	**Not present (ref) vs. present**	**2.556**	**1.227**	**5.325**	**0.012**	0.495	0.055	4.471	0.532
HIF‐1α cytoplasmic expression	**Ref = weak**	**1**			**0.009**	1			0.849
	**Moderate**	**4.111**	**1.655**	**10.212**	***0.002***	0.948	0.531	1.691	0.855
	**Strong**	**4.135**	**1.194**	**14.322**	**0.025**	1.269	0.420	3.838	0.673
HIF‐1α nuclear expression	Negative (ref) vs. positive	1.468	0.536	4.019	0.455	0.582	0.251	1.348	0.207
HIF‐1α nuclear percentage (%)		1.002	0.994	1.009	0.646	0.998	0.991	1.005	0.612
HIF‐2α cytoplasmic expression	**Ref = no expression + weak expression**	**1**			**<0.001**	1			0.822
	Moderate	1.620	0.902	2.909	0.106	0.933	0.538	1.621	0.807
	**Strong**	**4.022**	**2.128**	**7.603**	**<0.001**	0.841	0.463	1.526	0.569
HIF‐2α nuclear expression	Negative (ref) vs. positive	0.312	0.043	2.249	0.248	0.049	0.000	2777.745	0.588
HIF‐2α nuclear percentage (%)		0.865	0.637	1.174	0.352	0.298	0.004	23.849	0.588
PHD2 cytoplasmic expression	Ref = weak	1			0.437	1			0.175
	Moderate	1.263	0.544	2.933	0.588	6.282	0.860	45.873	0.070
	Strong	1.744	0.669	4.545	0.255	5.376	0.720	40.142	0.101
PHD2 nuclear expression	Negative (ref) vs. positive	0.164	0.040	0.678	*0.012*	0.379	0.234	0.612	<0.001
PHD2 nuclear percentage (%)		**0.988**	**0.981**	**0.996**	**0.002**	0.996	0.988	1.004	0.294
PHD3 cytoplasmic expression	Ref = weak	1			0.489	1			0.848
	Moderate	1.623	0.652	4.039	0.298	1.009	0.421	2.416	0.984
	Strong	1.952	0.638	5.975	0.241	1.131	0.439	2.915	0.799
PHD3 nuclear expression	Negative (ref) vs. positive	0.510	0.316	0.825	0.006	0.556	0.334	0.925	0.024
PHD3 nuclear percentage (%)		**0.983**	**0.975**	**0.991**	**<0.001**	0.987	0.967	1.007	0.190

**Multivariable COX model**		**HR**	**Lower 95% CI**	**Upper 95% CI**	***p*‐value**	**HR**	**Lower 95% CI**	**Upper 95% CI**	***p*‐value**
Age (years)		1.000	1.000	1.000	0.374				
pT‐stage	Ref = Stage T1				0.151				**0.023**
	Stage T2	2.293	0.920	5.714	0.075	**1.929**	**1.095**	**3.397**	**0.023**
	Stage T3‐4	2.026	0.831	4.938	0.120	0.468	0.088	2.503	0.375
Nuclear grade	Ref = Grade I				0.540				0.562
	Grade II	0.609	0.125	2.965	0.539	0.764	0.175	3.337	0.720
	Grade III	0.877	0.166	4.642	0.877	0.727	0.156	3.398	0.686
	Grade IV	1.149	0.180	7.338	0.883	1.441	0.255	8.137	0.679
Peripelvic fat invasion	Not present (ref) vs. present	0.942	0.473	1.875	0.865	**2.761**	**1.451**	**5.252**	**0.002**
Perirenal fat invasion	Not present (ref) vs. present	1.742	0.958	3.167	0.069				
Macrovascular invasion	Not present (ref) vs. present	0.835	0.330	2.113	0.703	1.703	0.336	8.631	0.520
Microvascular invasion	**Not present (ref) vs. present**	**2.789**	**1.190**	**6.539**	**0.018**	1.625	0.828	3.187	0.158
Tumor necrosis	Not present (ref) vs. present	1.270	0.705	2.289	0.426	**3.531**	**1.914**	**6.515**	**<0.001**
Sarcomatoid	Not present (ref) vs. present	1.336	0.504	3.538	0.560				
HIF‐1α cytoplasmic expression	**Ref = weak**				**0.010**				
	**Moderate**	**4.673**	**1.562**	**13.986**	**0.006**				
	Strong	2.020	0.362	11.254	0.423				
HIF‐2α cytoplasmic expression	Ref = weak				0.492				
	Moderate	0.892	0.462	1.724	0.734				
	Strong	1.360	0.602	3.075	0.459				
PHD2 nuclear expression	**Negative (ref) vs. positive**	**0.047**	**0.007**	**0.330**	**0.002**	**0.470**	**0.264**	**0.836**	**0.010**
PHD2 nuclear percentage (%)		1.001	0.989	1.013	0.916				
PHD3 nuclear expression	Negative (ref) vs. positive	0.594	0.287	1.229	0.160	1.033	0.579	1.842	0.913
PHD3 stained nuclei percentage (%)		0.992	0.978	1.007	0.316				

Abbreviations: CI, confidence intervals; HR, hazard ratio; Ref, reference.

Bold values were considered statistically significant at the *p* < 0.05 level.

In univariable analysis, positive nuclear expression of PHD2 and PHD3 were associated with decreasing risk of disease recurrence and death from RCC in both cohorts. In addition, higher pT‐stage, higher nuclear grade, the presence of peripelvic fat invasion, macrovascular and microvascular invasion, histological tumor necrosis and cytoplasmic expressions of HIF‐1α and HIF‐2α were significantly associated with a higher risk of disease recurrence or death from RCC in univariable analysis in both cohorts.

Clinical and histopathological features that had a significant association with RFS in the univariable analysis were included into the multivariable Cox model. Some of the clinical variables were significant in either, but not both, of the cohorts in multivariable analysis. Of the biomarkers, elevation in HIF1‐a cytoplasmic expression was significant in Helsinki cohort but not in Turku cohort. The only variable, including biomarkers and clinical parameters, that had significant HR in multivariable Cox analysis was PHD2 nuclear expression (positive vs. negative). For comprehensive Cox regression analysis results, see Table 3.

## 4 DISCUSSION

Members of the HIF pathway are known to be important regulators of malignant transformation and progression of ccRCC.^17^, ^18^ There are many prognostic studies about HIF‐1α and HIF‐2α but the prognostic role of PHDs and their cellular localization has remained less clear, which is why we focused on the results of PHDs in this study. Here, we studied the expression of PHD2 and PHD3 in tumor samples from patients who had undergone radical or PN for localized ccRCC and analyzed their associations with RFS in two independent patient cohorts. In this study, we observed that the absence of nuclear expression of PHD2 and PHD3 in cancer cells was associated with shorter RFS in the Helsinki training cohort as well as in the Turku validation cohort. Positive nuclear expression of PHD2 was associated with a decreased risk of disease recurrence or death from RCC in univariable analysis and retained its association in multivariable analysis regardless of other relevant histopathological prognostic factors such as pT‐stage, nuclear grade, microvascular invasion, and histological tumor necrosis in both cohorts. Higher percentages of cancer cells with positive nuclear PHD2 and PHD3 expression were also associated with lower pT‐stage and nuclear grade underlining the role of PHD2 and PHD3 mainly as a tumor suppressors as suggested earlier.^22^, ^23^

The findings of our study reveal positive nuclear PHD2 expression as a potential independent biomarker of favorable prognosis in patients with localized ccRCC. In other cancers, promising prognostic potential of PHD2 has been observed as high PHD2 levels have been reported to be associated with better prognosis at least in gastric cancer, breast cancer, and colorectal cancer.^29^, ^30^, ^31^ On the contrary, in head and neck squamous cell carcinomas, the nuclear translocation of PHD2 has been linked to a more aggressive phenotype.^30^ In univariate analysis, the nuclear expression of PHD3, in line with PHD2, was also associated with favorable RFS in both cohorts. In accordance with our findings, Kampantais et al. demonstrated that mRNA overexpression of PHD3 is inversely related to nuclear grade in RCC, but there has been a lack of evidence regarding the value of PHD3 in predicting treatment outcomes in ccRCC.^32^ The results of our study suggest that positive nuclear PHD3 expression could also be used as a favorable prognostic biomarker in patients with localized ccRCC.

Cytoplasmic expressions of PHD2 or PHD3 were not associated with RFS in either of the cohorts. This is in line with our previous study on PHD2 expression in HNSCC showing association of nuclear PHD2 rather than cytoplasmic PHD2 with tumor aggressiveness. The rationale behind this stronger effect of nuclear versus cytoplasmic expression must be studied further and the answer for this remains unknown.^33^ We observed that the cytoplasmic expression of PHD2 and PHD3 was accompanied by the cytoplasmic expression of HIF‐1α and‐2α, as expected, because their expression is elevated due to non‐functional VHL protein.^17^, ^21^ The evidence about the role of PHD2 and PHD3 in tumor development may seem controversial due to somewhat opposing effects observed in previous studies.^21^, ^22^, ^29^, ^34^ In this study, the nuclear percentage of HIF‐1α was inversely associated with the cytoplasmic expression of PHD2 and cytoplasmic expression of HIF‐2α was inversely associated with nuclear expression of PHD3 which is consistent with previous findings showing that PHD2 is the main regulator of HIF‐1α^22^, ^35^ and PHD3 is the main regulator of HIF‐2α.^20^, ^23^ However, in ccRCC one previous in vitro‐study has suggested that high PHD3 gene expression is needed to maintain high levels of HIF‐2α through regulation at mRNA level.^36^ In line, we observed high cytoplasmic HIF‐2α levels accompanied by high cytoplasmic PHD3 expression.

Besides HIF‐pathway, PHD2 and, in particular, PHD3 have been suggested to regulate several non‐HIF targets either in hydroxylase activity‐dependent or independent manner.^22^, ^23^, ^27^ PHD3 has been suggested to possess tumor suppression properties and to regulate the transcription of proteins involved in glucose metabolism, translational machinery, and proliferation in ccRCC cell lines apart from HIF pathway.^23^, ^37^ PHD2 overexpression has been shown to restrict tumor development regardless of HIF.^22^, ^38^ On the contrary, the silencing of PHD2 expression in a mouse osteosarcoma, lung carcinoma, and melanoma has been shown to restrict tumor growth suggesting PHD2 to be a potential target for anti‐tumoral therapy.^34^, ^39^ This oncogenic effect of PHD2 may be mediated by HIF‐independent mechanisms related to immune tolerance and vascular normalization and neovascularisation.^27^, ^34^, ^39^, ^40^ Further studies are warranted to fully understand the complex and controversial mechanisms of the HIF pathway proteins.

To our knowledge, this study is the first study with an external validation cohort evaluating the association of PHD2 and PHD3 expression with RFS in patients with localized ccRCC. The limitations of this study include its retrospective design which makes both cohorts vulnerable to selection bias and the limitations of reproducibility of IHC staining. Positive nuclear PHD2 expression was more applicable biomarker in the Turku validation cohort where 72.2% of tumors expressed positive nuclear PHD2 compared to 98.8% in the Helsinki cohort (*p* < 0.001). The ultimate reason for the difference is probably multivariable. In the Turku validation cohort, patients had more commonly pT1 and pT2 and low‐grade tumors, but also more commonly histological tumor necrosis compared to the Helsinki training cohort. Also, technical aspects on the processing of tumor samples, such as the formalin fixation time and the age of tissue block, could have affected the results of IHC stainings. However, the results on the association of positive nuclear PHD2 and PHD3 expression with longer RFS as well as lower pT‐stage and nuclear grade were similar in both cohorts supporting the generalizability of these results.

Moreover, our findings of the prognostic role of PHD2 and PHD3 in patients with localized ccRCC were in line with previous results observed in other cancer types on the prognostic value of HIF prolyl hydroxylases in cancer progression.^30^, ^31^, ^32^

## 5 CONCLUSIONS

According to this externally validated cohort study, the absence of nuclear HIF‐hydroxylases, PHD2 and PHD3, in cancer cells was associated with short RFS in patients with localized ccRCC. Moreover, positive nuclear expression of PHD2 predicted long RFS regardless of other relevant histopathological prognostic factors. The study suggests that nuclear PHD2 and PHD3 are potential prognostic biomarkers that could supplement traditional histopathological prognostic factors in patients with localized ccRCC. In conclusion, our novel PHD2/EGLN1 and PHD3/EGLN3 results warrant in‐depth mechanistic analysis. While there are certain limitations, primarily associated with sample size, the results are significant and suggest that these hydroxylases may play a crucial role in predicting the disease's course. Larger retrospective patient cohorts and prospective studies are needed to provide stronger evidence on the prognostic impact of PHD2 and PHD 3 in ccRCC.

## AUTHOR CONTRIBUTIONS

**Lassi Luomala:** Conceptualization (equal); data curation (lead); formal analysis (lead); funding acquisition (equal); investigation (lead); methodology (lead); resources (equal); software (equal); visualization (equal); writing – original draft (lead); writing – review and editing (lead). **Kalle Mattila:** Data curation (equal); investigation (equal); validation (equal); writing – original draft (supporting); writing – review and editing (equal). **Paula Vainio:** Data curation (equal); validation (equal); writing – review and editing (supporting). **Harry Nisén:** Conceptualization (equal); data curation (equal); resources (supporting); supervision (equal); writing – review and editing (supporting). **Teijo Pellinen:** Conceptualization (supporting); investigation (supporting); resources (supporting); software (supporting); writing – review and editing (supporting). **Jouni Lohi:** Conceptualization (supporting); data curation (supporting); investigation (supporting); writing – review and editing (supporting). **Teemu D. Laajala:** Software (supporting); visualization (lead); writing – review and editing (supporting). **Petrus Järvinen:** Conceptualization (equal); data curation (supporting); methodology (supporting); project administration (supporting); writing – review and editing (supporting). **Anna‐Riina Koskenniemi:** Data curation (supporting); writing – review and editing (supporting). **Panu Jaakkola:** Conceptualization (equal); investigation (equal); methodology (equal); project administration (supporting); supervision (lead); validation (equal); writing – original draft (supporting); writing – review and editing (equal). **Tuomas Mirtti:** Conceptualization (lead); data curation (equal); formal analysis (supporting); funding acquisition (lead); investigation (equal); methodology (equal); project administration (lead); resources (supporting); software (supporting); supervision (lead); validation (equal); writing – original draft (supporting); writing – review and editing (equal).

## FUNDING INFORMATION

This work was supported by Kidney Foundation (Munuaissäätiö), the Finnish Urological Association, Diagnostic Center (Helsinki University hospital) and the Cancer Foundation Finland.

## ETHICS STATEMENT

The study was approved by the Institutional Review Boards of Helsinki and Turku University Hospitals: The Ethical Committee of Helsinki University Hospital (diary number HUS/1040/2018), the hospital study permit from the corresponding unit head (HUS/419/2018), and Turku University Hospital License number T06/032/15. Informed consent was waived due to the retrospective design of the study according to Finnish legislation on the secondary use of health data. Data were anonymized before statistical analyses and handled in a manner that met general regulations on data protection.

## Supporting information


Appendix S1.

## Data Availability

The data that support the findings of this study are available from the corresponding author, [L.L], upon reasonable request. Restrictions and additional conditions may apply to the availability of these data from the Helsinki University Hospital.
