# Supplementary material for: Low nuclear expression of HIF‐hydroxylases PHD2/EGLN1 and PHD3/EGLN3 are associated with poor recurrence‐free survival in clear cell renal cell carcinoma
Source: Cancer Med. 2024 Feb 24;13(3):e6998. doi: 10.1002/cam4.6998 (PMC10891444; doi:10.1002/cam4.6998)
Supplement: Supplementary file 1 — Appendix S1. [file CAM4-13-e6998-s001.docx]

Supporting information


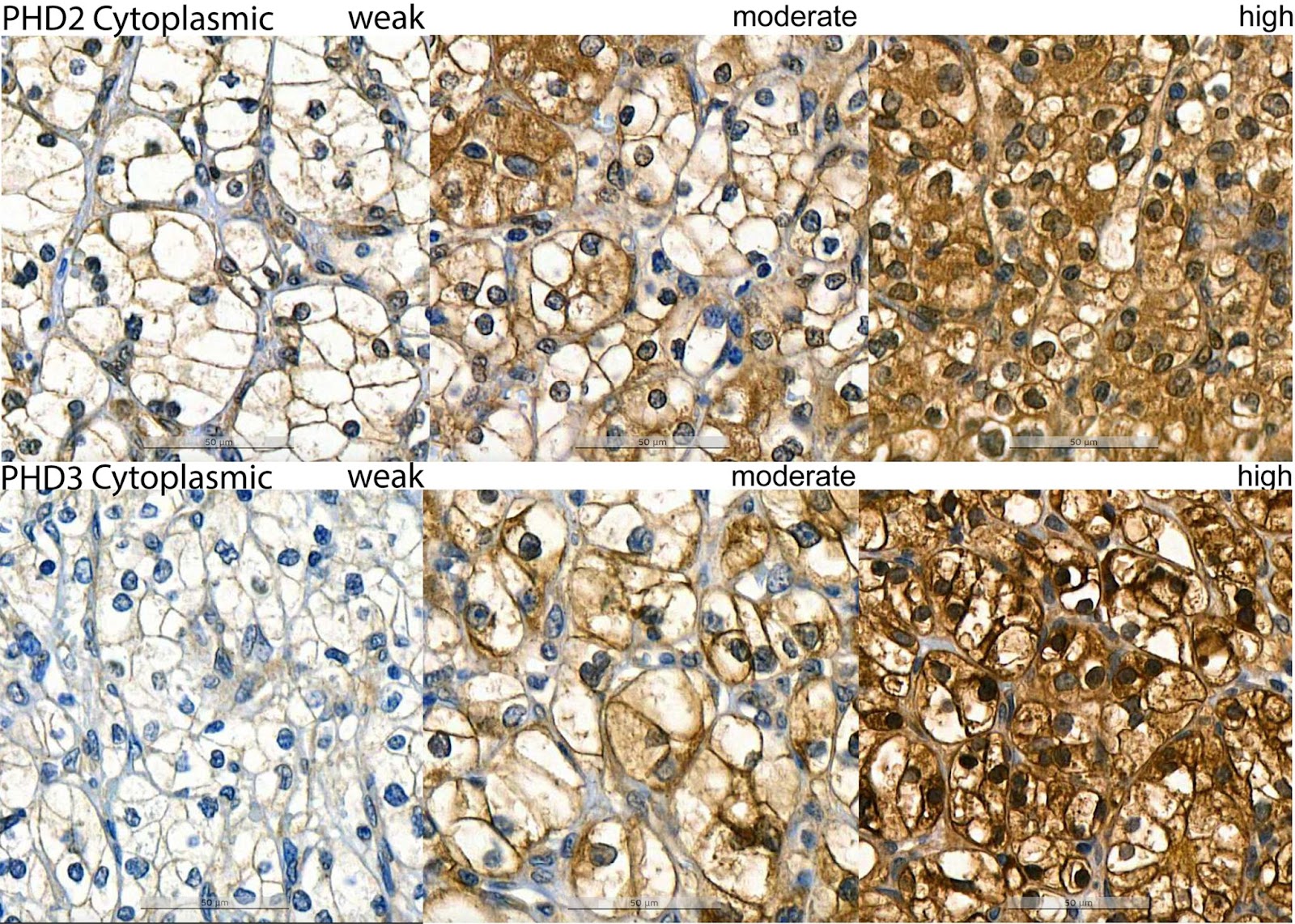


**Supplementary figure 1.** Examples of cytoplasmic expression of PHD2 and PHD3.


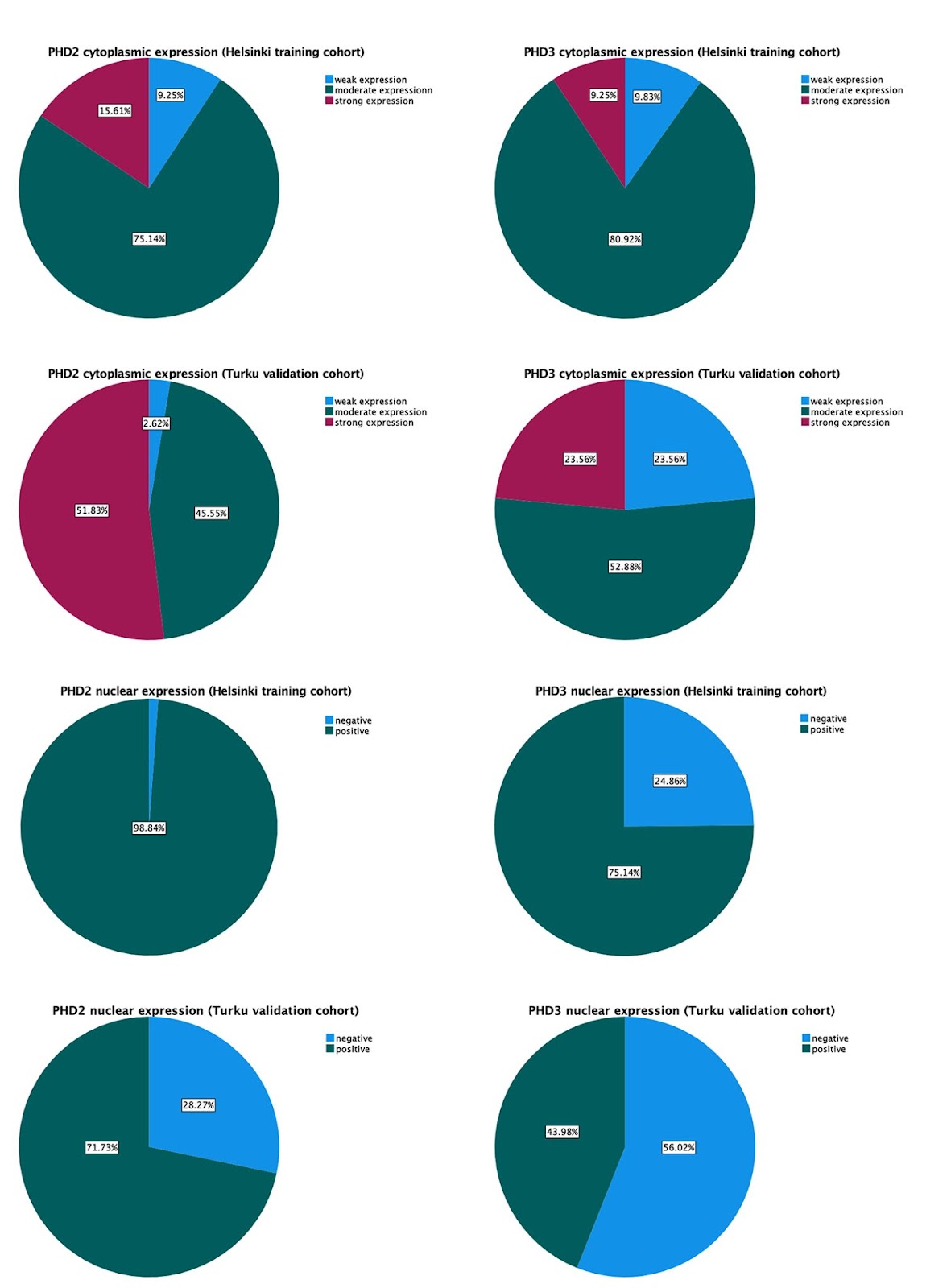

**Supplementary figure 2.** Distributions of PHD2 and PHD3 expressions in training (Helsinki) and validation (Turku) cohorts.

|  | **HIF-1α (Helsinki training cohort)** | **HIF-1α (Turku validation cohort)** | **HIF-2α (Helsinki training cohort)** | **HIF-2α (Turku validation cohort)** |
| --- | --- | --- | --- | --- |
| **Cytoplasmic expression** | 100 % | 100 % | 93.6 % | 99.5 % |
| **Nuclear expression** | 93.1 % | 94.8 % | 3.5 % | 0.5 % |
| **Mean nuclear percentage (IQR)** | 73.53 % (52.5–90 %) | 67.2 % (45–95 %) | 0.74 % (0–0) | 0.013 % (0–0) |

**Supplementary table 1.** Expression of HIF-1α and HIF-2 α in the Helsinki and Turku cohorts


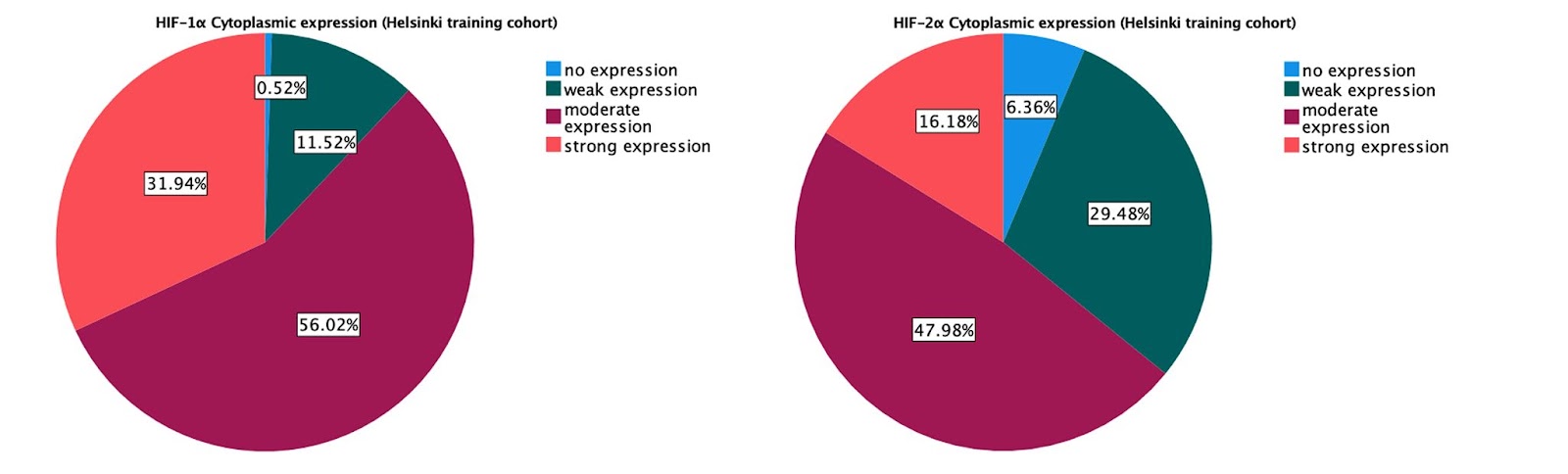


**Supplementary figure 3.** Distributions of HIF1-α and HIF-2α cytoplasmic expression.

**
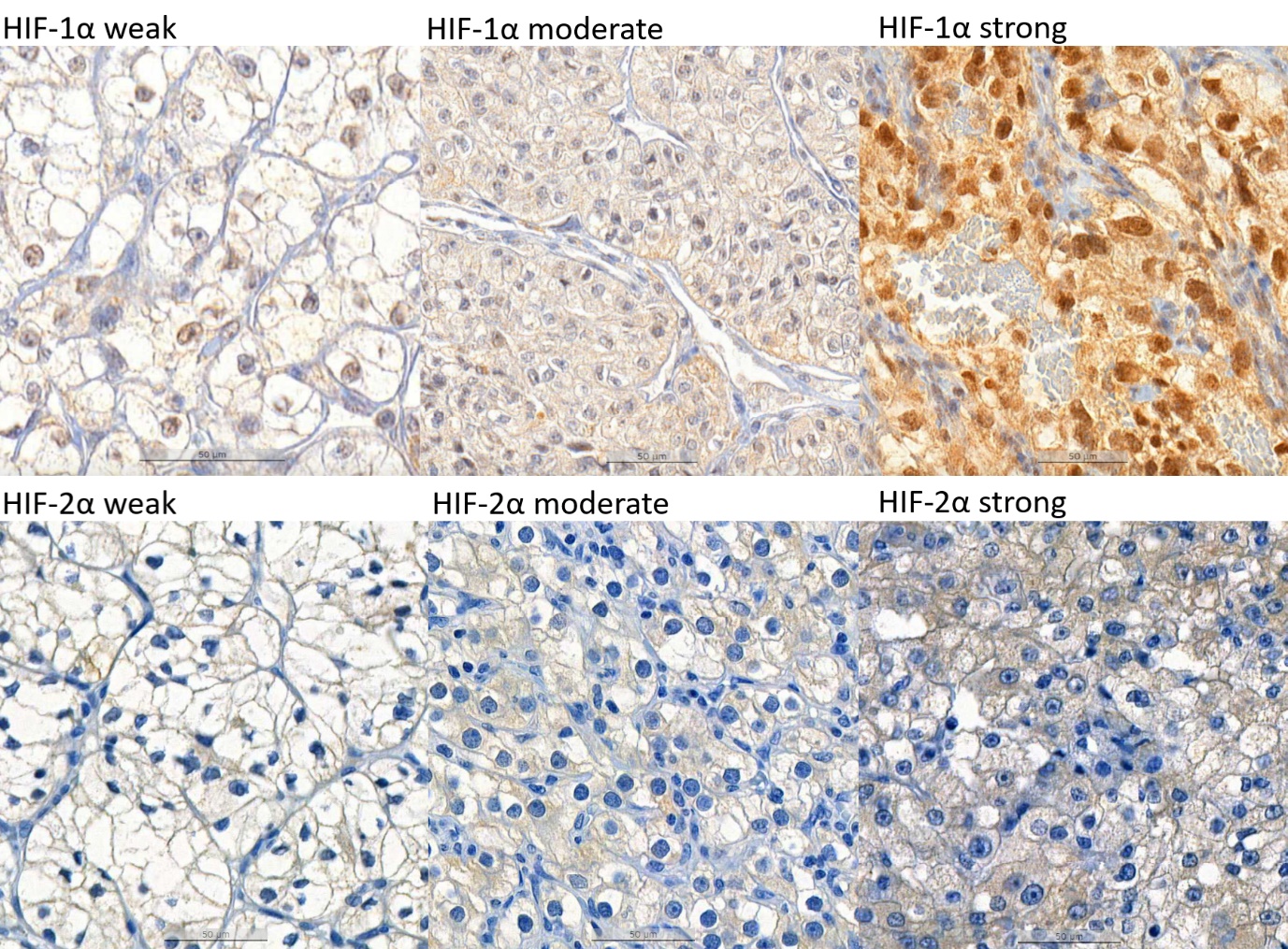
**

**Supplementary figure 4.**  Examples of HIF-1α and HIF-2α cytoplasmic expression.

| **Significant associations between HIF pathway biomarkers and clinical parameters in Helsinki and Turku cohorts** | | |
| --- | --- | --- |
|  | **Helsinki training cohort** | **Turku validation cohort** |
| **PHD2 nuclear percentage** | **Coefficient** | **Coefficient** |
| pT stage | -.315** | -.251** |
| Fuhrman grade | -.189* | -.290** |
| PHD3 cytoplasm/membrane | .220** | .210** |
| PHD3 nuclear percentage | .687** | .500** |
| HIF-1a nuclear percentage | .598** | .467** |
| HIF-2a cytoplasm/membrane | -.163* | -.230** |
| **PHD3 nuclear percentage** | **Coefficient** | **Coefficient** |
| pT stage | -.412** | -.153* |
| Fuhrman grade | -.404** | -.185* |
| PHD2 cytoplasm/membrane | -.168* |  |
| PHD2 nuclear percentage | .687** | .500** |
| HIF-2a cytoplasm/membrane | -.272** |  |
| HIF-1a nuclear percentage | .496** | .406** |
| **PHD2 cytoplasm/membrane** | **Coefficient** | **Coefficient** |
| Fuhrman grade | .195** | .343** |
| PHD3 cytoplasm/membrane | .244** | .292** |
| PHD3 nuclear percentage | -.168* |  |
| HIF-2a cytoplasm/membrane | .371** | .577** |
| HIF-1a cytoplasm/membrane | .302** | .432** |
| HIF-1a nuclear percentage | -.159* | -.145* |
| **PHD3 cytoplasm/membrane** | **Coefficient** | **Coefficient** |
| PHD2 cytoplasm/membrane | .244** | .292** |
| PHD2 nuclear percentage | .220** | .210** |
| HIF-2a cytoplasm/membrane | .214** | .258** |
| HIF-1a cytoplasm/membrane | .360** | .302** |
| HIF-1a nuclear percentage | .259** | .233** |
| **HIF-1a cytoplasm/membrane** | **Coefficient** | **Coefficient** |
| PHD2 cytoplasm/membrane | .302** | .158* |
| PHD3 cytoplasm/membrane | .360** | .302** |
| HIF-2a cytoplasm/membrane | .363** | .403** |
| **HIF-1a nuclear percentage** | **Coefficient** | **Coefficient** |
| pT stage | -.180* |  |
| PHD2 cytoplasm/membrane | -.159* | -.145* |
| PHD2 nuclear percentage | .598** | .467** |
| PHD3 cytoplasm/membrane | .259** | .233** |
| **HIF-2a cytoplasm/membrane** | **Coefficient** | **Coefficient** |
| pT stage | .243** |  |
| Fuhrman grade | .287** | .232** |
| PHD2 cytoplasm/membrane | .371** | .577** |
| PHD2 nuclear percentage | -.163* | -.230** |
| PHD3 cytoplasm/membrane | .214** | .258* |
| PHD3 nuclear percentage | -.272** |  |
| HIF-1a cytoplasm/membrane | .363** | .403** |
| **HIF-2a nuclear percentage** | **Coefficient** | **Coefficient** |
| pT stage | -.195** | -.057 |

**Supplementary table 2.** Significant associations between HIF-markers and clinical parameters in Helsinki training cohort and Turku validation cohort calculated by Pearson correlation coefficient.

*p<0.05, **p<0.001.


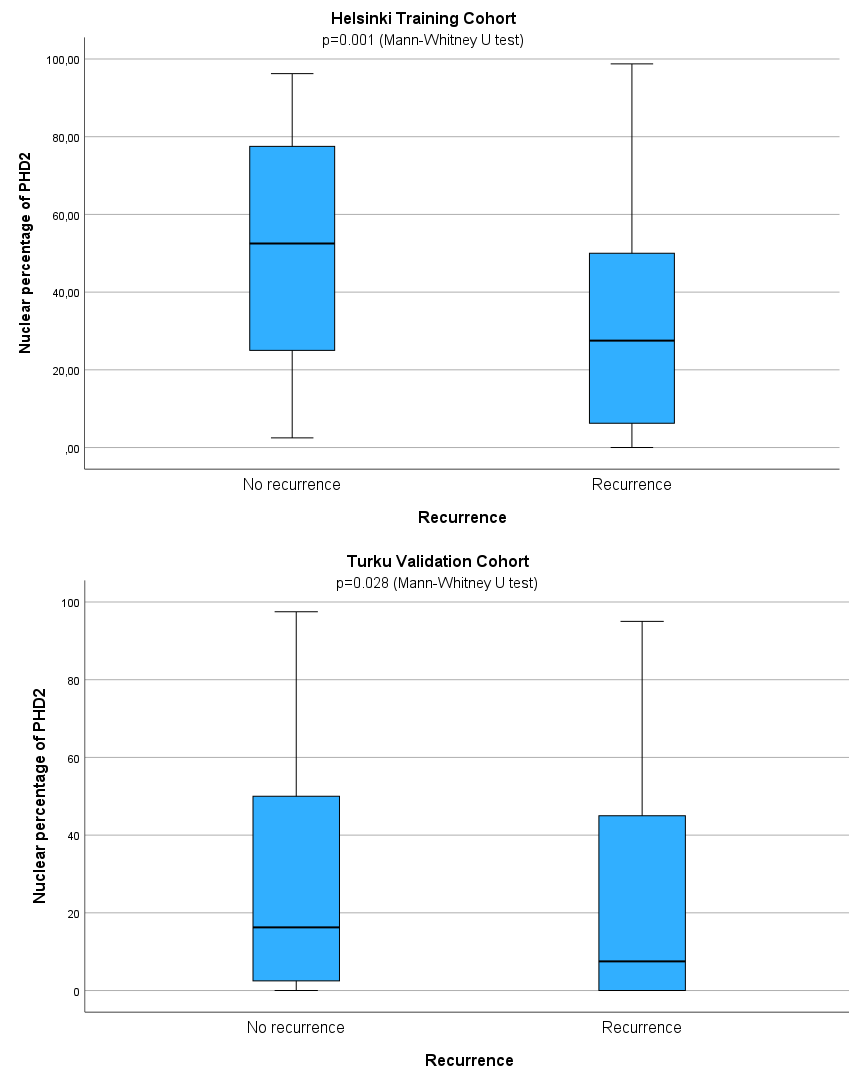


**Supplementary figure 5.** Nuclear percentage of PHD2 in patients with or without recurrence. Significance presented as two-sided *P*-values (Mann-Whitney U test).


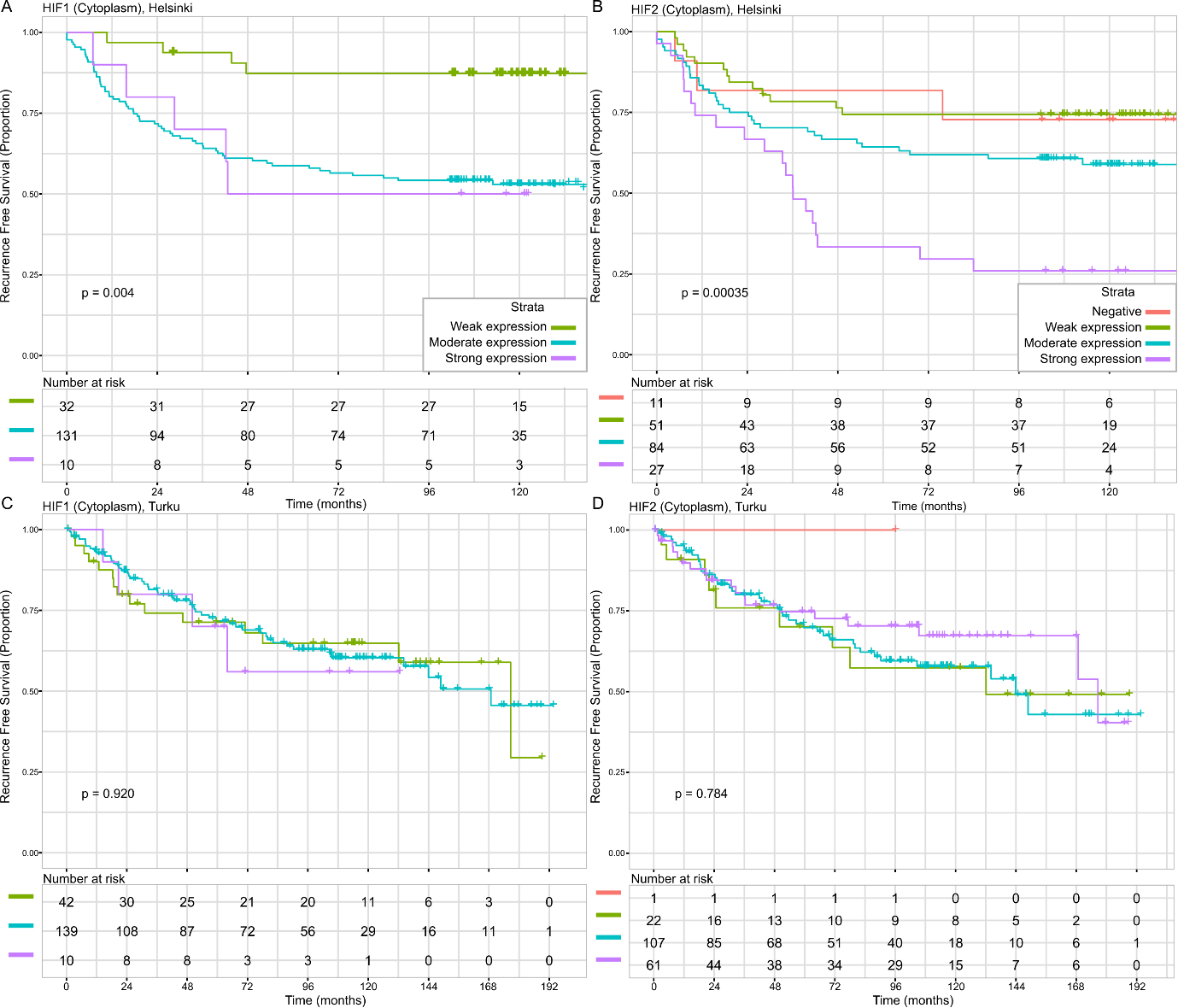


**Supplementary figure 6.** Kaplan-Meier survival analyses for probability of disease recurrence or death from RCC in the Helsinki training cohort and Turku validation cohort. HIF1 cytoplasmic expression (A and C) and HIF2 cytoplasmic expression (B and D). *P*-values are from a two-sided log-rank (Mantel-Cox) test.
